# Nitrogen Availability Affects the Metabolic Profile in Cyanobacteria

**DOI:** 10.3390/metabo11120867

**Published:** 2021-12-14

**Authors:** Kosuke Inabe, Ayaka Miichi, Mami Matsuda, Takanobu Yoshida, Yuichi Kato, Ryota Hidese, Akihiko Kondo, Tomohisa Hasunuma

**Affiliations:** 1 Innovation and Technology and Engineering Biology Research Center, Kobe University, Nada, Kobe 657-8501, Japan; inabe@people.kobe-u.ac.jp (K.I.); yuichi@shark.kobe-u.ac.jp (Y.K.); akondo@kobe-u.ac.jp (A.K.); 2 Graduate School of Science, Innovation and Technology, Kobe University, Nada, Kobe 657-8501, Japan; ayaka.miichi@gmail.com (A.M.); matsuda_mami@harbor.kobe-u.ac.jp (M.M.); t-yoshida@port.kobe-u.ac.jp (T.Y.); hidese@people.kobe-u.ac.jp (R.H.); 3 Department of Chemical Science and Engineering, Graduate School of Engineering, Kobe University, Nada, Kobe 657-8501, Japan

**Keywords:** cyanobacteria, nitrogen, metabolome, photosynthesis

## Abstract

Nitrogen is essential for the biosynthesis of various molecules in cells, such as amino acids and nucleotides, as well as several types of lipids and sugars. Cyanobacteria can assimilate several forms of nitrogen, including nitrate, ammonium, and urea, and the physiological and genetic responses to these nitrogen sources have been studied previously. However, the metabolic changes in cyanobacteria caused by different nitrogen sources have not yet been characterized. This study aimed to elucidate the influence of nitrate and ammonium on the metabolic profiles of the cyanobacterium *Synechocystis* sp. strain PCC 6803. When supplemented with NaNO_3_ or NH_4_Cl as the nitrogen source, *Synechocystis* sp. PCC 6803 grew faster in NH_4_Cl medium than in NaNO_3_ medium. Metabolome analysis indicated that some metabolites in the CBB cycle, glycolysis, and TCA cycle, and amino acids were more abundant when grown in NH_4_Cl medium than NaNO_3_ medium. ^15^N turnover rate analysis revealed that the nitrogen assimilation rate in NH_4_Cl medium was higher than in NaNO_3_ medium. These results indicate that the mechanism of nitrogen assimilation in the GS-GOGAT cycle differs between NaNO_3_ and NH_4_Cl. We conclude that the amounts and biosynthetic rate of cyanobacterial metabolites varies depending on the type of nitrogen.

## 1. Introduction

Nitrogen is an important element for living things, being used in amino acids, nucleotides, lipids, and sugars, which are building blocks of all forms of life. Cyanobacteria are Gram-negative bacteria and are known to be oxygenic photosynthetic microorganisms that utilize solar energy to generate chemical energy (ATP and NADPH). Such chemical energy is used in the Calvin–Benson–Bassham cycle (CBB cycle), glycolysis, and tricarboxylic acid (TCA cycle). Similar to other organisms, non-diazotrophic cyanobacteria, such as *Synechocystis* sp. PCC 6803 can take up nitrogen as NO_3_^−^, NO_2_^−^, NH_4_^+^, CO(NH_2_)_2_ (urea), and arginine [1,2]. Diazotrophic cyanobacteria (for example, *Anabaena* sp. PCC 7120) can fix N_2_ in heterocyst cells using nitrogenase [3]. Cyanobacteria require reducing power when using the former nitrogen sources (NO_3_^−^ and NO_2_^−^), but not when using the latter (NH_4_^+^, urea, and arginine). NO_3_^−^ and NO_2_^−^ transporters are already identified as NrtA-D. NH_4_^+^ is transported by ammonium transporters Amt1-3 and urea by urea transporters UrtA-E [4,5,6]. NO_3_^−^ in the cell is reduced to NO_2_^−^ by ferredoxin-nitrate reductase, NarB, and finally reduced to NH_4_^+^ by NirA [7,8]. Urea in the cell is converted by UreA-B to NH_3_ [6,9]. NH_4_^+^ is produced from arginine by arginine dihydrolase [2]. The GS (glutamine synthetase)-GOGAT (glutamate synthase or glutamine-oxoglutarate cycle amido transferase) cycle synthesizes glutamate and glutamine from NH_4_^+^ and 2OG [10]. The resulting Glu is mainly consumed as a nitrogen source.

Although cyanobacteria can assimilate nitrogen compounds as mentioned above, the choice of nitrogen sources is important for cultivation because photosynthesis and growth are affected by the type of nitrogen source [11,12,13]. In addition, there are differences in gene expression associated with utilizing the nitrogen sources and changes in activity of enzymes involved in nitrogen assimilation when cyanobacteria are exposed to each of the nitrogen sources, NO_3_^−^, NH_4_^+^, and urea [14,15,16]. The physiological response of cyanobacteria to nitrogen sources also varies according to the species [13,17]. We previously reported that when *Synechocystis* sp. PCC 6803 was phototrophically cultivated in the presence of NH_4_Cl, it produced a higher content of intracellular organic acids including malate, fumarate, and succinate under dark anoxic fermentation than cells grown in NaNO_3_, resulting in increased succinate secretion [18]. In this way, the influences of different types of nitrogen sources have been investigated to reveal the physiological responses of cyanobacteria and their application. However, metabolites produced with different types of nitrogen sources have not been fully investigated, in contrast to the effects of nitrogen starvation [19,20]. Moreover, in our previous report, we revealed that the different metabolic profiles produced during dark anoxic cultivation after a transfer from phototrophic cultivation with different nitrogen sources enhanced the production of succinate. These findings prompted us to investigate the metabolic profiles under phototrophic conditions.

In this study, we aimed to clarify the metabolic responses to different nitrogen sources. For this purpose, we performed a combination of in vivo ^15^N-labeling of metabolites and metabolome analysis. The ^15^N-labeling technique is applied to detect metabolites of interest in cyanobacteria or to examine the metabolic behavior of a few metabolites [2,17,21,22]. This technique enables us to compare the metabolic turnover under different nitrogen sources by calculating the ^15^N labeling rate at each time point. Using this technique and metabolome analysis, we compared the metabolic profiles and synthesis rates of amino acids in *Synechocystis* sp. PCC 6803 when grown in NaNO_3_ or NH_4_Cl, revealing distinct metabolic profiles between the different nitrogen sources.

## 2. Results

### 2.1. Growth in Different Types of Nitrogen Source

**Figure 1:**
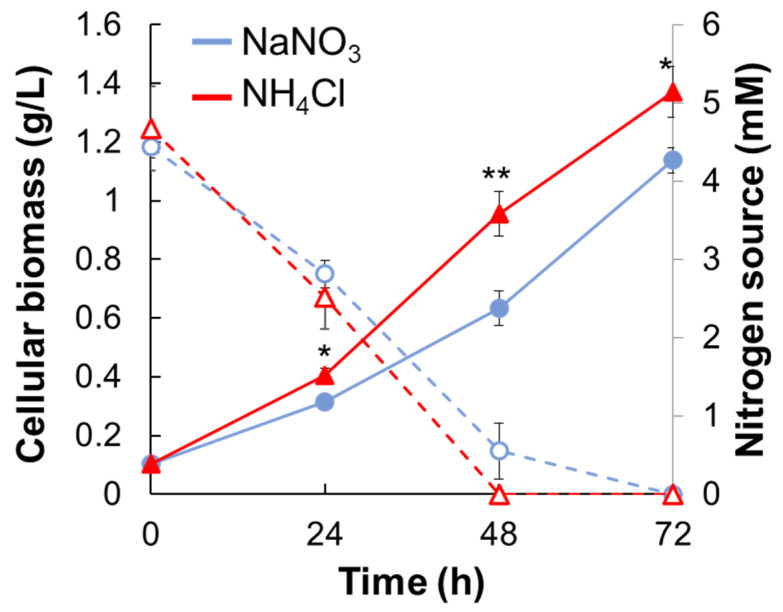
Cell growth of *Synechocystis* and the residual nitrogen concentration under different types of nitrogen. The cell growth of *Synechocystis* cultivated in NaNO_3_ medium or NH_4_Cl medium under phototrophic conditions was compared. Blue lines, NaNO_3_ medium; red lines, NH_4_Cl medium; solid lines, the cellular biomass (dried cell weight); dotted lines, the residual nitrogen concentration in the medium. Error bars indicate the standard deviation of three replicate experiments. Statistical significance was determined using Welch’s *t*-test (* <0.05, ** <0.01).

*Synechocystis* sp. PCC 6803 (hereafter *Synechocystis*) was cultivated in BG11 medium with 5 mM NaNO_3_ or NH_4_Cl (hereafter NaNO_3_ medium or NH_4_Cl medium) under phototrophic growth conditions (Figure 1 and Figure S1). The growth rate of *Synechocystis* was also calculated based on the growth data by 48 h when there are the residual nitrogen sources. The growth rate of *Synechocystis* grown in NaNO_3_ medium or NH_4_Cl medium was 0.028 ± 0.002 h^−1^ and 0.036 ± 0.002 h^−1^. The growth rate of *Synechocystis* in NH_4_Cl medium was significantly faster than that in NaNO_3_ medium throughout the cultivation. In contrast, there was no significant difference in the residual amounts of NaNO_3_ and NH_4_Cl in either medium.

### 2.2. Metabolome Analysis with Different Types of Nitrogen Source

**Figure 2:**
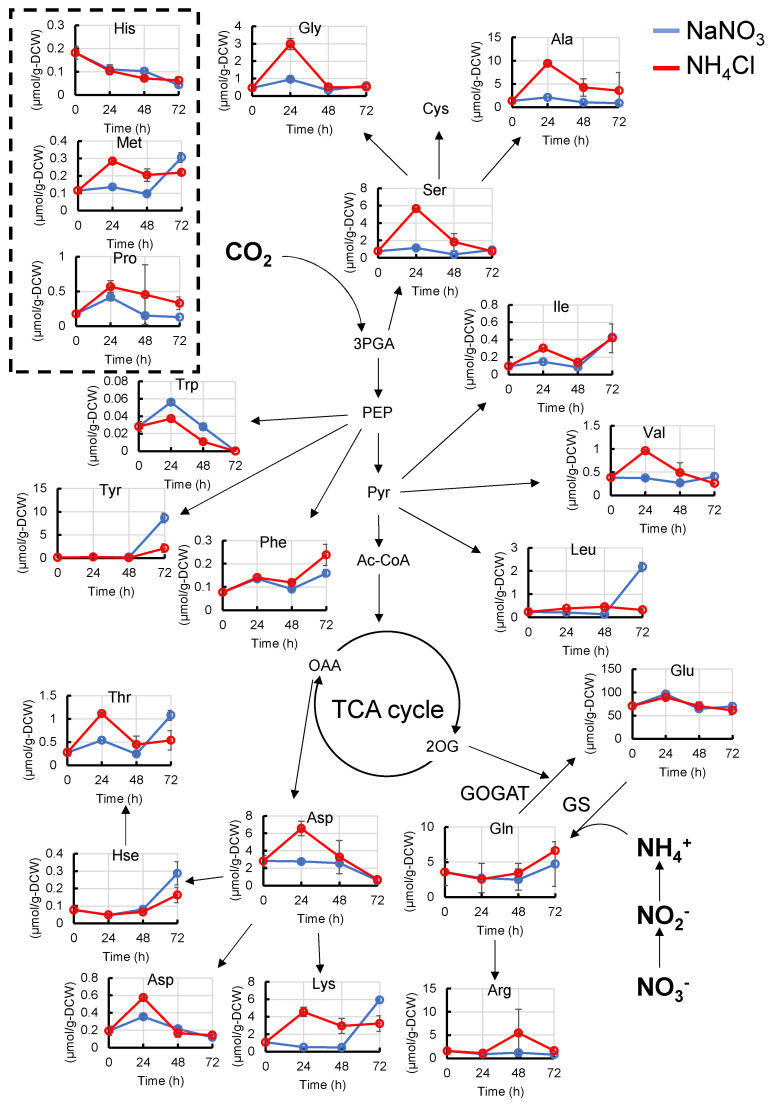
Metabolic profiles (amino acids) of *Synechocystis* cultured with NaNO_3_ or NH_4_Cl. The abundance of amino acids when *Synechocystis* was cultivated in NaNO_3_ medium or NH_4_Cl medium were compared at each time point. Error bars indicate the standard deviation of three replicate experiments. Blue lines, cultivation in NaNO_3_ medium; red lines, cultivation in NH_4_Cl medium. 3PGA, 3-phosphoglycerate; Ac-CoA, acetyl-CoA; Ala, alanine; Arg, arginine; Asp, aspartate; Asn, asparagine; Cys, cysteine; Glu, glutamate; Gln, glutamine; His, histidine; Hse, homoserine; Ile, isoleucine; Leu, leucine; Met, methionine; PEP, phosphoenolpyruvate; Phe, phenylalanine; Pro, proline; Pyr, pyruvate; Ser, serine; Thr, threonine; Trp, tryptophan; Tyr, tyrosine; Val, valine.

As nitrogen is thought to be assimilated mainly through the GS-GOGAT cycle, which synthesizes glutamate (Glu) and glutamine (Gln) from NH_4_^−^ and 2OG, we first compared the amino acid levels with different nitrogen sources (Figure 2 and Figure S2). The pool sizes of serine (Ser), glycine (Gly), threonine (Thr), alanine (Ala), aspartate (Asp), asparagine (Asn), lysine (Lys), valine (Val), and isoleucine (Ile) when grown in NH_4_Cl medium were higher than those in NaNO_3_ medium 24 h after the start of cultivation. The pool sizes of methionine (Met) were still high 48 h after the start of cultivation. The pool sizes of Gln and Glu were the same between the NaNO_3_ and NH_4_Cl media. On the other hand, the pool size of tryptophan (Trp) when grown in NH_4_Cl medium was lower than that in NaNO_3_. Since Thr and Lys are synthesized from Asp, the pool sizes of Thr and Lys increased with an increase in Asp.

**Figure 3:**
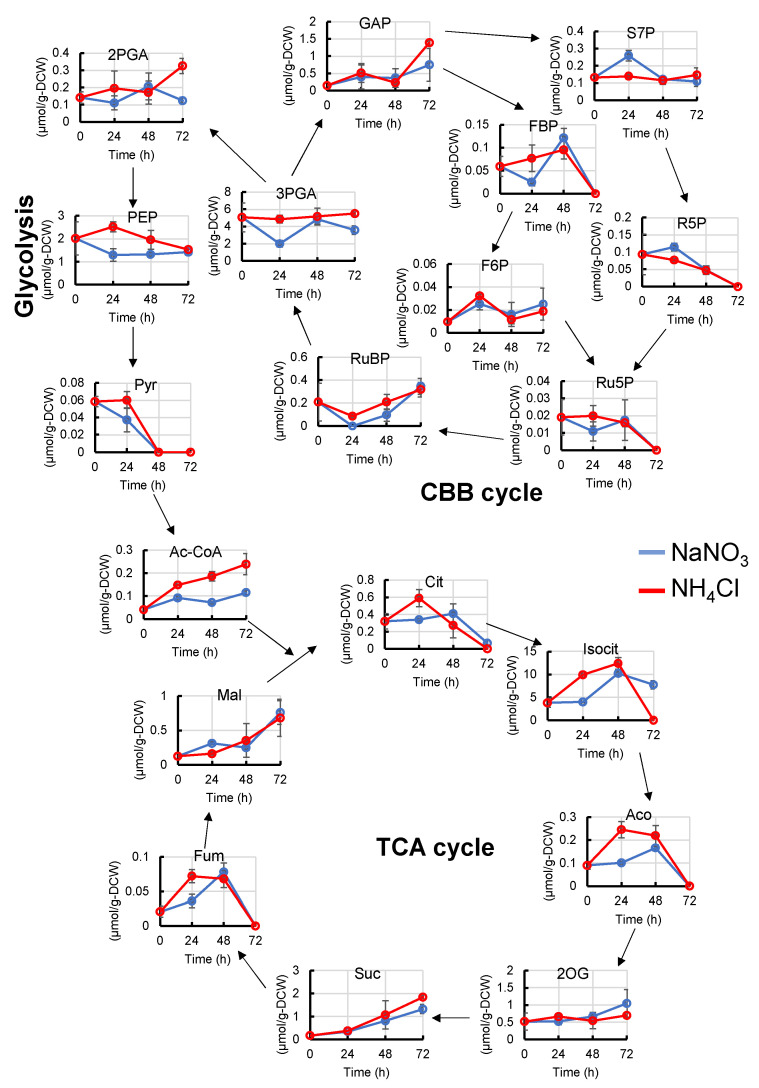
Metabolic profiles (CBB cycle, glycolysis and TCA cycle) of *Synechocystis* cultured with NaNO_3_ or NH_4_Cl. The abundance of some metabolites in the CBB cycle, glycolysis, and TCA cycle were compared at each time point. Error bars indicate the standard deviation of three replicate experiments. Blue lines, cultivation with NaNO_3_; red lines, cultivation with NH_4_Cl. 2PGA, 2-phosphoglycerate; 2OG, 2-oxoglutarate; 3PGA, 3-phosphoglycerate; Ac-CoA, acetyl-Coenzyme A; Cit, Citrate; F6P, fructose 6-phosphate; FBP, fructose 1,6-phosphate; Fum, fumarate; GAP, glyceraldehyde 3-phosphate; Isocit, isocitrate; PEP, phosphoenolpyruvate; Pyr, pyruvate; R5P, ribose 5-phosphate; Ru5P, ribulose 5-phosphate; RuBP, ribulose 1,5-bisphosphate; S7P, sedoheptulose 7-phosphate; Suc, succinate.

To understand why the differences in amino acid levels occur, we also examined some metabolites of the CBB cycle, glycolysis, and TCA cycle (Figure 3). After 24 h from the start of cultivation, the levels of ribulose-1,5-bisphosphate (RuBP), 3-phosphoglycerate (3PGA), phosphoenolpyruvate (PEP), acetyl-coenzyme A (Ac-CoA), citrate (Cit), aconite (Aco), isocitrate (Isocit), and fumarate (Fum) were higher, and sedoheptulose 7-phosphate (S7P) and ribose 5-phosphate (R5P) were lower than those with NaNO_3_. In contrast, no difference was observed in the pool size of pyruvate (Pyr), although PEP, Ile, Val, and acetyl-CoA were increased. We speculate that Pyr synthesized from PEP may be immediately converted to Val, Ile, and Ac-CoA.

### 2.3. ^15^N-Turnover Analysis with Different Types of Nitrogen

**Figure 4:**
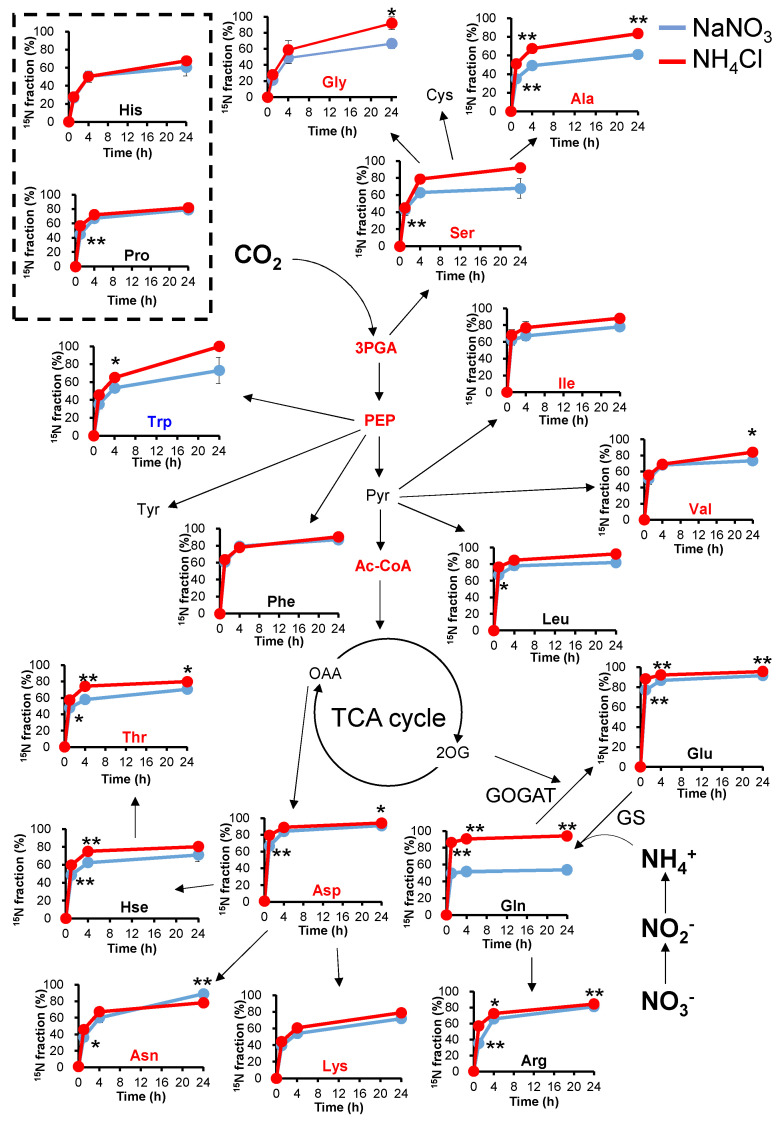
Analysis of ^15^N turnover under different nitrogen conditions. *Synechocystis* was cultivated in Na^15^NO_3_ or ^15^NH_4_Cl media after cell inoculation. The ^15^N labeling rates of amino acids and their related metabolites were compared at each time point. Values represent the mean ± standard deviation of three independent experiments. Blue lines, cultivation with NaNO_3_; red lines, cultivation with NH_4_Cl. Red and blue characters indicate higher and lower amounts of metabolites when grown in NH_4_Cl. Statistical significance was determined using Welch’s *t*-test (* <0.05, ** <0.01). 3PGA, 3-phosphoglycerate; Ac-CoA, acetyl-coenzyme A; Ala, alanine; Arg, arginine; Asp, aspartate; Asn, asparagine; Cys, cysteine; Glu, glutamate; Gln, glutamine; His, histidine; Hse, homoserine; Ile, isoleucine; Leu, leucine; Met, methionine; PEP, phosphoenolpyruvate; Phe, phenylalanine; Pro, proline; Pyr, pyruvate; Ser, serine; Thr, threonine; Trp, tryptophan; Tyr, tyrosine; Val, valine.

The growth rates and pool sizes of the metabolites were different in NaNO_3_ and NH_4_Cl. However, the nitrogen flow in the cell when grown in NaNO_3_ or NH_4_Cl media remains unclear. To reveal nitrogen flow with different nitrogen sources, we measured the time-resolved labeling rate of amino acids by ^15^N stable isotope labeling (Figure 4 and Figure S3). For this purpose, *Synechocystis* cells were taken after 24 h of cultivation and transferred to fresh BG11 medium containing ^15^NH_4_Cl or Na^15^NO_3_ (labeling time = 0 h). The labeling rates of Ala, Ser, and Gly, which are synthesized from 3PGA, were significantly higher in NH_4_Cl medium than in NaNO_3_ medium. The larger pool sizes of Ala, Ser, and Gly when *Synechocystis* are grown in NH_4_Cl medium might result from the higher ^15^N labeling rate. The labeling rate of Glu and Gln were significantly higher in NH_4_Cl medium than in NaNO_3_ medium, which is consistent with a previous report [17]. However, the pool sizes of Glu and Gln did not change, as shown in Figure 2. This indicates that when *Synechocystis* is grown in NaNO_3_ medium, one of the two nitrogen atoms in Gln is ^14^NH_4_^+^, which can result from cellular nitrogen sources such as amino acids.

### 2.4. ^15^N Labelling Rate and Order in Glutamine Synthesis

**Figure 5:**
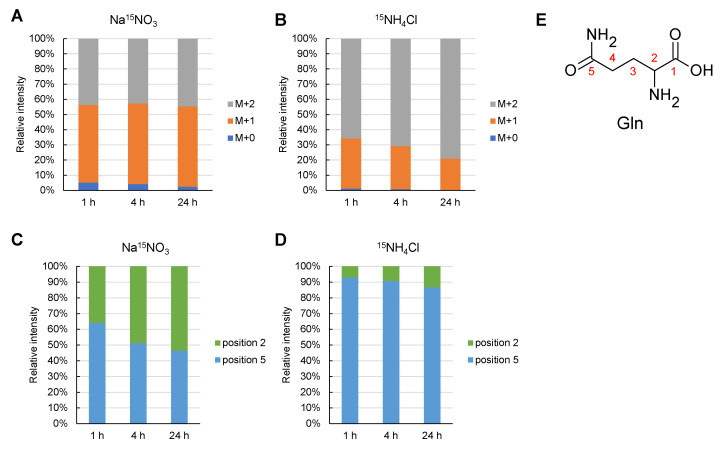
^15^N labelling rate and its position in Gln. The ^15^N labeling rate and its position in Gln (glutamine) were examined by LC-MS/MS. A and B indicate the number of the ^15^N-labelled NH_2_ group in glutamine and ^15^N-labelled rate in each ^15^N-labelled number of all Gln. (**A**), Cultivation in Na^15^NO_3_ medium; (**B**), Cultivation in ^15^NH_4_Cl medium. (**C**,**D**) indicate the position of the two NH_2_ groups in Gln when one of the two was labeled at M^+1^ in (**A**–**C**), Cultivation in Na^15^NO_3_ medium; (**D**), Cultivation in ^15^NH_4_Cl medium. (**E**), the position number in Gln, as indicated in (**C**,**D**).

To elucidate the mechanism underlying the lower labeling rate of glutamine in the NH_4_Cl medium, the position of the ^15^N-labeled nitrogen atom in the Gln molecule was examined using liquid chromatography-tandem mass spectrometry with the multiple reaction monitoring method (LC-MS/MS MRM) (Figure 5). ^15^N labeling conditions were the same as those for the experiments performed in Figure 4 (see Materials and Methods). The ^15^N labeling rate of Gln when cultured in Na^15^NO_3_ was almost constant for 24 h after *Synechocystis* inoculation (Figure 5A,B). On the other hand, when cultured in ^15^NH_4_Cl, the rate of ^15^N labeling of one of the two nitrogen atoms in Gln gradually decreased, and the unlabeled rate of Gln was 0.23% (2.5% in the presence of Na^15^NO_3_). As shown in Figure 4, the total ^15^N labeling rate of the two nitrogen atoms in Gln was 71% in Na^15^NO_3_ and 89% in ^15^NH_4_Cl. Next, the position of the ^15^N-labelled nitrogen atom in glutamine was investigated. The positions of the ^15^N labeling are described as positions 2 and 5, which are the amino groups of the main and side chains (Figure 5E). Position 5 of Gln was preferentially labeled 1 h after *Synechocystis* was inoculated under culture conditions with Na^15^NO_3_. After an interval of 24 h, the labeling rates of positions 2 and 5 became equal. On the other hand, in ^15^NH_4_Cl medium, position 5 was always preferentially labeled during the period of 24 h after *Synechocystis* inoculation.

### 2.5. Nitrogen Assimilation Rate by Glutamine Synthase and Glutamate Dehydrogenase

**Figure 6:**
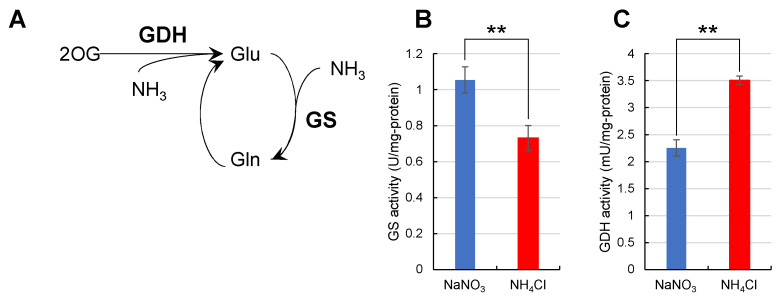
Catalytic activity of GS and GDH in whole cell lysates. The catalytic activity of GS and GDH in whole cell lysates of *Synechocystis* grown in different nitrogen sources was examined. Values represent the mean ± standard deviation of three independent experiments. (**A**), Reaction scheme for each enzyme. (**B**), GS activity when grown in NaNO_3_ or NH_4_Cl media. (**C**), GDH activity when grown in NaNO_3_ or NH_4_Cl media. GS, glutamine synthase; GDH, glutamate dehydrogenase. Statistical significance was determined using Welch’s *t*-test (** <0.01).

From these results, the enzymatic activity for Gln synthesis was assumed to be affected by the type of nitrogen source. To test this hypothesis, we measured the catalytic activities of glutamine synthetase (GS) and glutamate dehydrogenase (GDH) from whole-cell lysates (Figure 6). The catalytic activity of GS was 1.1 U/mg-protein when *Synechocystis* was grown in NaNO_3_ medium and 0.73 U/mg-protein when grown in NH_4_Cl medium. In a previous report, the expression level of GS was higher in NaNO_3_ medium than in NH_4_Cl medium [14]. When considered with the results from this previous report, the difference in the catalytic activity of GS in Figure 6B reflects the difference in the expression level of GS itself. On the other hand, the activity of GDH was 3.5 mU/mg-protein when *Synechocystis* was grown in NH_4_Cl medium and 2.3 mU/mg-protein in NaNO_3_ medium; the catalytic activity of GDH in NH_4_Cl was higher than that in NaNO_3_.

## 3. Discussion

### 3.1. Different Assimilation Mechanisms for Nitrogen Depending on the Nitrogen Source

In this study, we wished to elucidate the mechanisms by which *Synechocystis* grown under different nitrogen sources assimilates nitrogen, and more generally, to clarify how it responds to different nitrogen sources. Different types of nitrogen altered the growth rate, pool sizes of metabolites, and nitrogen assimilation rate of *Synechocystis* (Figure 1, Figure 2, Figure 3, Figure 4 and Figure 5). In addition, the catalytic activities of GS and GDH were different for different types of nitrogen sources (Figure 6).

**Figure 7:**
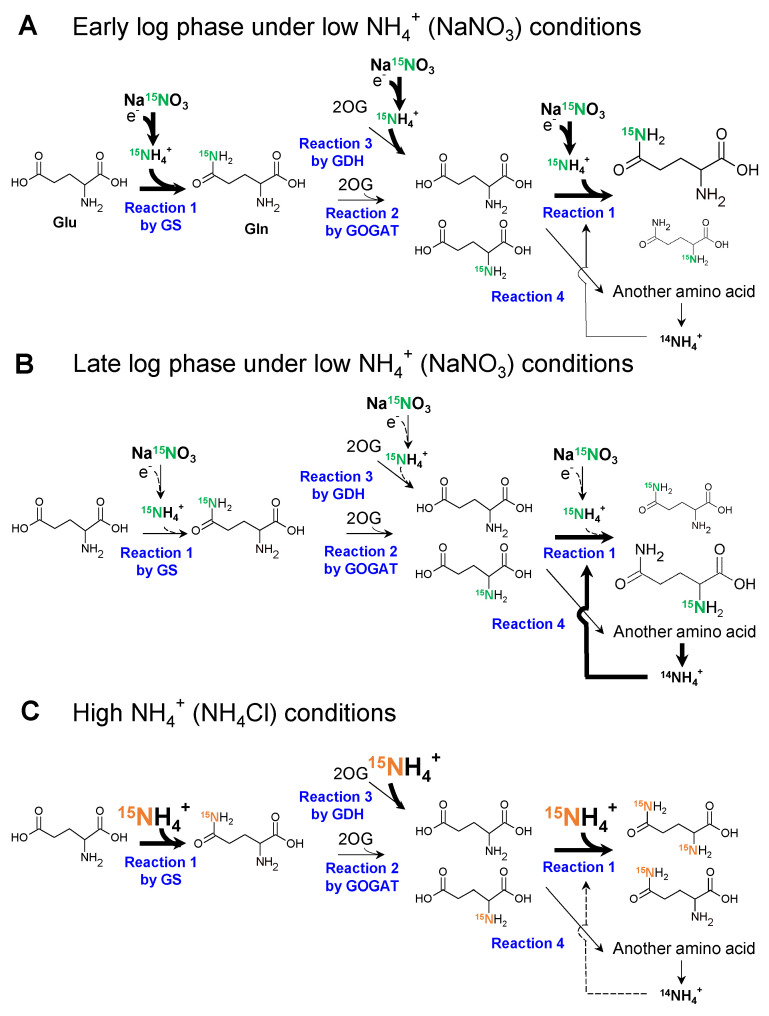
Reaction scheme with different nitrogen sources. The reaction scheme around the GS-GOGAT cycle and the GDH pathway was constructed based on the results obtained. The bold arrows, thin arrows, and dotted arrows indicate the enhanced, not changed, and weakened fluxes in each reaction. (**A**), the reaction scheme during the early log phase when *Synechocystis* was grown in Na^15^NO_3_ medium; (**B**), the reaction scheme during late log phase when *Synechocystis* was grown in Na^15^NO_3_ medium; (**C**), the reaction scheme when *Synechocystis* was grown in ^15^NH_4_Cl medium. The enlarged characters and molecular structure represent larger pool sizes of the molecule.

Based on the findings of this study, we propose a model for nitrogen assimilation by *Synechocystis* grown in different nitrogen sources (Figure 7). The model was prompted by ^15^N labeling experiments. In contrast to the assimilation of NH_4_^+^, *Synechocystis* was unable to assimilate NO_3_^−^ by itself and required an additional reducing power to convert it to NH_4_^+^ (Figure 7A). This means that the reduction of NO_3_^−^ appears to be the rate-limiting step for nitrogen assimilation. Therefore, at the beginning of the log phase, when there is sufficient photosynthetic reducing power, GS synthesizes Gln with ^15^NH_4_^+^, resulting in the rapid labeling of the side chain of Gln with ^15^N (Reaction 1 in Figure 7A) [23]. This hypothesis is consistent with the finding that the preferentially ^15^N-labeled nitrogen was in position 5 until 1 h later (Figure 5C). Subsequently, the ^15^N labelled NH_2_ group is rapidly transferred to 2OG by GOGAT, resulting in two Glu molecules (Reaction 2 in Figure 7A) [24]. GS can also synthesize doubly ^15^N-labeled Gln from newly reduced ^15^NH_4_^+^ and ^15^N-labeled Glu (Reaction 1 in Figure 7A). Furthermore, ^15^N-labeled Glu can be used as a nitrogen source in another pathway to generate another amino acid (Reaction 4 in Figure 7A). However, the reducing power gradually decreased as the light transmittance of the culture medium decreased during the transition from the early log phase (Figure 7A) to the late log phase (Figure 7B). It has been previously shown that light is necessary to reduce NO_3_^−^, and Fd, which accepts the reducing power from the photosystem, reduces NO_3_^−^ and NO_2_^−^ to NH_4_^+^ [7,8,23,24]. Thus, GS cannot use the newly reduced ^15^NH_4_^+^ but reuses ^14^NH_4_^+^, which derived from amino acids due to the decrease in light (Figure 7B). As a result, Reaction 1 with ^15^NH_4_^+^ in Figure 7 would not occur. In fact, the ^15^N labeling rate of position 2 in Figure 5C gradually increased and became equal to that of position 5 after 24 h. This can also explain why the ^15^N labeling rate of Gln was 50% (Figure 4) and 71% (Figure 5A).

In contrast to NO_3_^−^, reducing power is not required to assimilate NH_4_^+^. Figure 5D illustrates that position 5 in Gln is always dominant throughout the 24 h period, and Figure 5B indicates that 90% of nitrogen in all Gln molecules was labeled. The light conditions cannot be the rate-determining step to assimilate NH_4_^+^, and Reaction 1 in Figure 7C continues unless NH_4_^+^ is lacking. Therefore, we assumed that the ^15^N labeling rate quickly approached 90%, as shown in Figure 4 and Figure 5B.

We also considered the possibility that the higher labeling rate with ^15^NH_4_Cl in Figure 4 and Figure 5 resulted from the higher activity of the GDH pathway, as shown in Figure 6C. However, such possibilities can be excluded because, in Figure 5D,E, position 2 should be preferentially labeled if the GDH pathway is activated when *Synechocystis* is cultivated in NH_4_Cl medium. GDH synthesizes Glu from 2OG and NH_4_^+^, suggesting that position 5 is not labeled [25] and GS activity was much higher than the activity of GDH (Figure 6).

The catalytic activity of GS was about 1000-fold higher than that of GDH in Figure 6B,C. For *Synechocystis*, GDH is not essential for growth under the high CO_2_ conditions [26]. In *Escherichia coli*, GDH supplies glutamine in the absence of a carbon source because GDH can produce it without ATP originating from carbon utilization [27]. This means that the high CO_2_ conditions which were adopted in our experiments might have caused the 1000-fold change differences between the catalytic activity of GS and GDH.

In this study, we found that the type of nitrogen sources can affect the metabolic profile of *Synechocystis*. The altered metabolic profile and labeling rate of *Synechocytstis* in Figure 2, Figure 3, Figure 4 and Figure 5 when *Synechocystis* was grown in NaNO_3_ or NH_4_Cl media came from the availability of nitrogen. When *Synechocystis* was grown in NH_4_Cl medium, NH_4_^+^ could be used without limitation unless NH_4_Cl was absent in the medium. On the other hand, additional reducing power is required to use NaNO_3_ because *Synechocystis* cannot use NO_3_^−^ directly. This means that the amount of light transmitted, which is the source of reducing power, can be the rate-determining step. Thus, *Synechocystis* switch the main nitrogen sources from the external to internal nitrogen sources which arise from internal amino acids or proteins to produce glutamine during the change of growth stage when grown in NaNO_3_ medium. This switch between external and internal nitrogen sources might enable *Synechocystis* to grow at the constant rate. When grown in NH_4_Cl medium, the residual additional reducing power for *Synechocystis* to assimilate nitrogen enables it to supply reducing power to other pathways. The pool size of 3PGA increased and that of S7P and R5P decreased, as shown in Figure 3. The altered pool sizes of these metabolites might be caused by the enhanced CBB cycle, consuming the residual additional reducing power that was not used in the reduction of NO_3_^−^. Accordingly, the abundance of some metabolites in glycolysis and TCA cycle, and amino acids including 3PGA itself increased in Figure 2 and Figure 3. We suspect the high cell growth rate was accomplished by higher abundance of those metabolites.

### 3.2. The Choice of Nitrogen Source: NO_3_^−^ or NH_4_^+^?

So far, we revealed that *Synechocystis* switches the main nitrogen sources from the external to internal nitrogen sources during the change of growth stage when grown in NaNO_3_ medium and does not need to switch the nitrogen sources in NH_4_Cl medium. This speculation suggests that NH_4_^+^ is ideal nitrogen sources for *Synechocystis*. However, there is a side effect of using NH_4_^+^ as a nitrogen source. The electron transport rate in *Synechocystis* decreased in the presence of over 15 mM NH_4_Cl according to a previous report [11]. This means that excess NH_4_^+^ can potentially inhibit photosynthesis in *Synechocystis*. In addition, GS-GOGAT cycle also requires the reducing power to assimilate nitrogen sources [1]. If the amount of NH_4_^+^ supplied exceeds the reducing power from photosynthesis, the accumulation of NH_4_^+^ would inhibit photosynthesis.

On the other hand, it is confirmed that there was no influence on photosynthesis over 15 mM NaNO_3_ in the same previous report. We guess the reason the excess amount of NH_4_^+^ cannot accumulate in the cell because the production of NH_4_^+^ is dependent on the availability of reducing power arisen from photosynthesis. This means that all the nitrogen assimilation pathway is dependent on the photosynthesis when grown in NaNO_3_, and it is beneficial for *Synechocystis* to perform the nitrogen assimilation in concert with photosynthesis and cell growth, preventing the excess accumulation of NH_4_^+^ in the cell.

The proper type of nitrogen depends on the species of cyanobacteria, given that they can assimilate various types of nitrogen sources, such as NO_3_^−^ and NH_4_^+^, as shown in this paper. NH_4_^+^ stimulated the growth of *Synechocystis,* as shown in Figure 1. In contrast, it attenuated the growth of *Arthrospira* (*Spirulina*) sp. [12,16] and did not affect the growth of *Microcystis aeruginosa* NIES-843 [17]. The reasons for the differing responses of cyanobacteria species to different types of nitrogen sources remain unclear. However, we revealed that the enhancement of metabolite pool size and nitrogen turnover by NH_4_^+^ stimulated the growth of *Synechocystis* in this study. A comparison of the comprehensive metabolic profiles of these cyanobacteria species under different types of nitrogen might reveal the reasons for the different responses of cyanobacteria species.

## 4. Materials and Methods

### 4.1. Strain and Cultivation Conditions

*Synechocystis* sp. PCC 6803 strain was pre-cultivated in BG11 medium containing 20 mM HEPES-KOH (pH 7.7) and 17.6 mM NaNO_3_ under 50 μmol photons m^−2^ s^−1^ at 30 °C for 4 days, as described previously [18]. After pre-cultivation, *Synechocystis* was inoculated into modified BG11 medium containing 50 mM HEPES-KOH (pH 7.7) and 5 mM NaNO_3_ or NH_4_Cl (NaNO_3_ medium or NH_4_Cl medium) and cultivated under 1% (*v*/*v*) CO_2_ and 100 μmol photons m^−2^ s^−1^ at 30 °C. The culture medium was recovered at the indicated times described below for further analysis. Statistical analysis was conducted using Welch’s *t*-test (* <0.05, ** <0.01).

### 4.2. Measurement of the Intracellular Metabolite Concentration

Extraction and analysis of the intracellular metabolites was performed as previously reported [18]. The procedure is described briefly. For the analysis of the intracellular metabolite concentration, the culture medium corresponding to 5 mg of dry cell weight was recovered at 0, 24, 48, and 72 h after inoculation with *Synechocystis*. After filtration, the collected cells were washed with 20 mM (NH_4_)_2_CO_3_. The intracellular metabolite was extracted using pre-cooled methanol containing the internal standard, and the water-soluble phase was collected by mixing chloroform. The soluble protein was removed by filtration, and the resultant water phase containing the metabolite was evaporated under vacuum. The dried metabolites were dissolved in pure water and subjected to CE-MS analysis.

### 4.3. ^15^N-Metabolic Turnover Analysis

The assimilation ratio of newly added nitrogen sources at each time point was determined using stable isotope ^15^N-labelled Na^15^NO_3_ or ^15^NH_4_Cl. *Synechocystis* was transferred to the modified BG11 medium containing 5 mM Na^15^NO_3_ or ^15^NH_4_Cl 24 h after inoculation and cultivated under 1% (*v*/*v*) CO_2_ and 100 μ mol photons m^−2^ s^−1^ at 30 °C. The culture medium corresponding to 5 mg of dry cell weight was recovered at 0, 4, and 24 h, and the intracellular metabolites were analyzed by CE-MS as described above or by LC-MS/MS MRM. The procedure of sample preparation was the same as described above Section 4.2 (measurement of the intracellular metabolite concentration) and LC-MS/MS MRM analysis was performed by employing Nexera X2 high-performance liquid chromatography system and a LCMS-8060 triple quadrupole mass spectrometer (Shimadzu Corporation, Kyoto, Japan), as described previously [28]. The ^15^N labeling rate was calculated as performed in ^13^C labeling experiments in previous reports [18]. The relative isotopomer abundance (*m_i_*) for each metabolite in which the *i*^15^N atoms were incorporated is calculated as follows:

(1) $$m_{i}\left(\%\right)=\frac{M_{i}}{\sum_{j=0}^{n}M_{j}}\times 100$$

(2) N15 fraction(%)=∑i=1ni×min 

where *M_i_* represents the isotopomer abundance of metabolite incorporating *i*^15^N atoms, and *n* is the number of nitrogen atoms in the metabolite. Statistical analysis was conducted using Welch’s *t*-test (* <0.05, ** <0.01).

### 4.4. Enzymatic Assay of Whole Cell Lysate

*Synechocystis* cells that were cultured in the presence of NaNO_3_ or NH_4_Cl were collected by centrifugation (3000× *g*, 4 °C, 10 min) and washed with nitrogen-free BG11 medium. The cells were collected by centrifugation and resuspended in 60 mM HEPES-KOH (pH 7.0). The suspended cells were disrupted by sonication, and the cell debris was removed by centrifugation (20,000× *g*, 4 °C, 20 min). After centrifugation, the supernatant was collected, and the protein concentration was determined using the Takara BCA Protein assay kit (Takara, Shiga, Japan). Glutamine synthetase (GS) assays were performed as described previously with some modifications [15]. Fifteen microliters of whole cell lysate containing 16 μg of protein was mixed well with 185 μL reaction solution 1 containing 60 mM HEPES-KOH (pH 7.0), 40 mM glutamine, 4 mM MnCl_2_, 60 mM hydroxylamine, 1 mM ADP, and 20 mM sodium arsenate. Reaction solution 1 was incubated at 30 °C for 20 min and terminated by the addition of an equal volume of FeCl_3_ solution containing 0.5 M HCl, 247 mM FeCl_3_, and 196 mM trichloroacetate. GS activity (units/mg protein) was calculated at an absorption wavelength of 500 nm to determine the amount of γ-glutamylhydroxamate. The glutamate dehydrogenase (GDH) assay was performed as described previously with some modifications [29]. Whole cell lysate containing 0.24 mg of protein was mixed well with 875 μL reaction solution 2 containing 85 mM Tris-HCl (pH 8.0), 10 mM 2OG, and 50 mM NH_4_Cl. The catalytic reaction of GDH was initiated by the addition of 50 μL of 0.2 mM NADPH, and the decrease in NADPH at absorption at 340 nm was monitored using a spectrophotometer V-670 (JASCO, Tokyo, Japan) to calculate GDH activity (mUnits/mg-protein). Statistical analysis was conducted using Welch’s *t*-test (* <0.05, ** <0.01).

## Data Availability

Data is contained within the article or Supplementary Material.

## Supplementary Materials

The following are available online at https://www.mdpi.com/article/10.3390/metabo11120867/s1, Figure S1: Growth curve of *Synechocystis* during 168 h in NaNO_3_ medium or NH_4_Cl medium under phototrophic conditions, Figure S2: Comparison of metabolic profiles in the Urea cycle. The pool sizes of some metabolites of the Urea cycle were examined. Blue lines, cultivation with NaNO_3_; Red lines, cultivation with NH_4_Cl.; Arg, Arginine; CPS, Carbamoyl phosphate; Glu, Glutamate; Orn, Ornithine, Figure S3: ^15^N labelling rates of metabolites of the Urea cycle, ^15^N labelling rates of the Urea cycle was compared at each time point. Blue lines, cultivation with NaNO_3_; Red lines, cultivation with NH_4_Cl.; Arg, Arginine; CPS, Carbamoyl phosphate; Glu, Glutamate; Orn, Ornithine.
