# Supplementary material for: Nitrogen Availability Affects the Metabolic Profile in Cyanobacteria
Source: Metabolites. 2021 Dec 14;11(12):867. doi: 10.3390/metabo11120867 (PMC8707274; doi:10.3390/metabo11120867)
Supplement: Supplementary file 1 [file metabolites-11-00867-s001.zip › metabolites-1514767-supplementary.pdf]

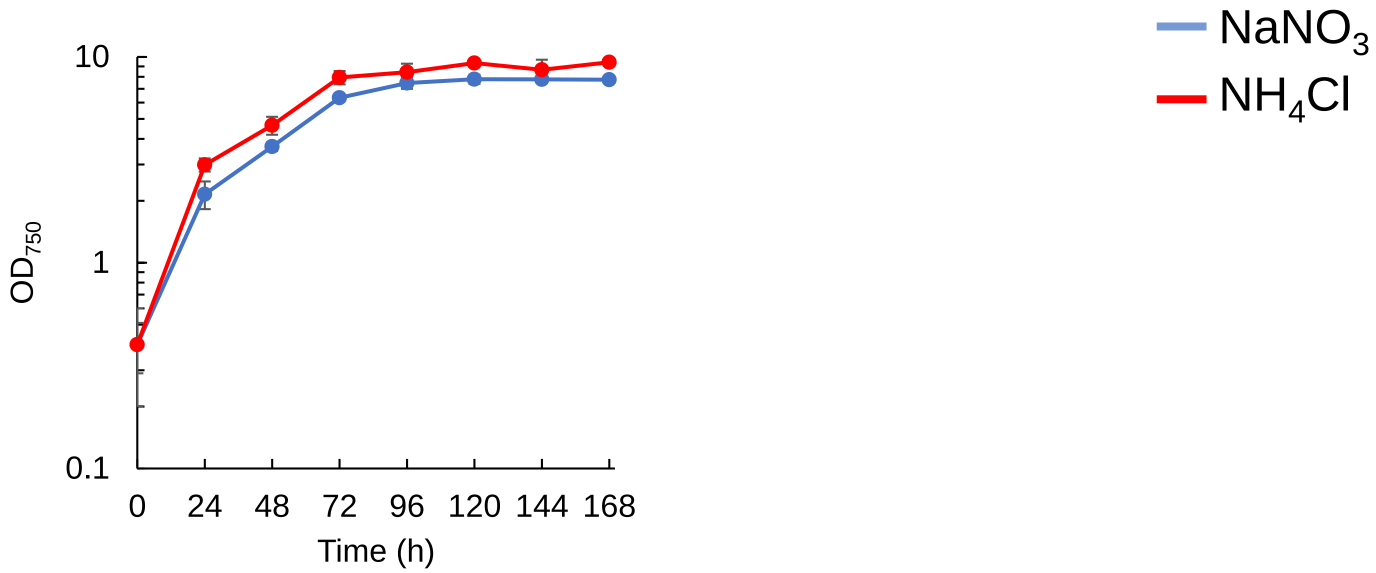

Figure S1 Growth curve of *Synechocystis* during 168 h in $NaNO_3$ medium or NH_4Cl medium under phototrophic conditions

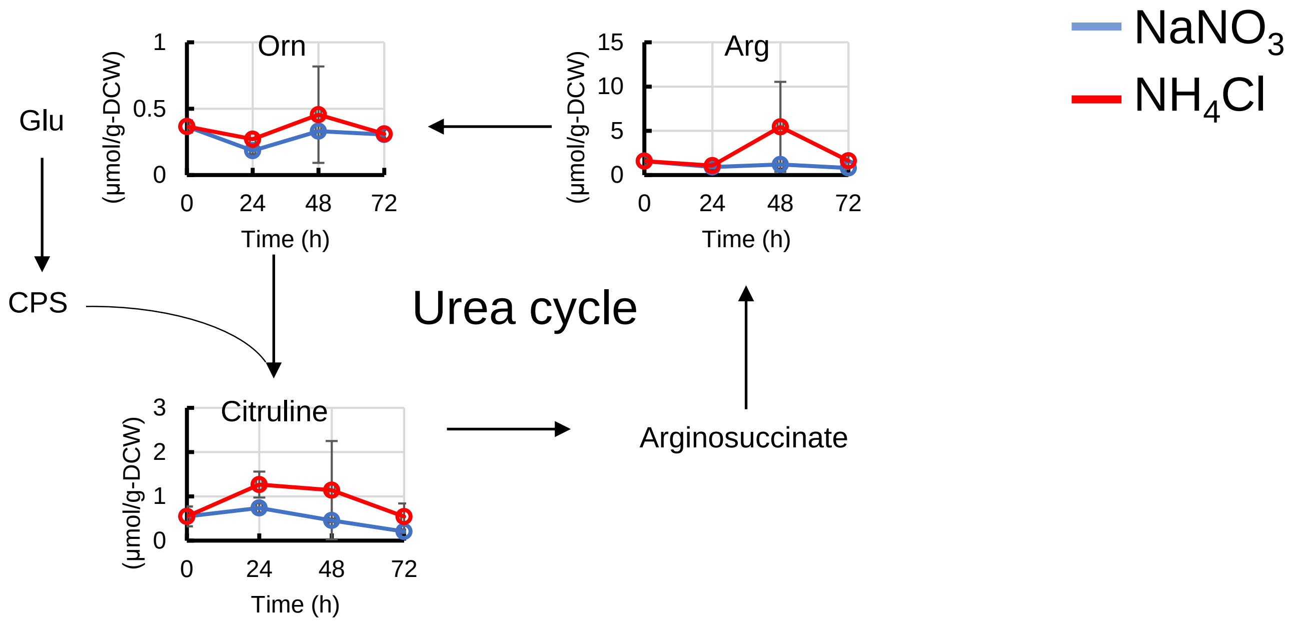

Figure S2 Comparison of metabolic profiles in the Urea cycle. The pool sizes of some metabolites of the Urea cycle were examined. Blue lines, cultivation with $NaNO_3$; Red lines, cultivation with NH_4Cl.; Arg, Arginine; CPS, Carbamoyl phosphate; Glu, Glutamate; Orn, Ornithine

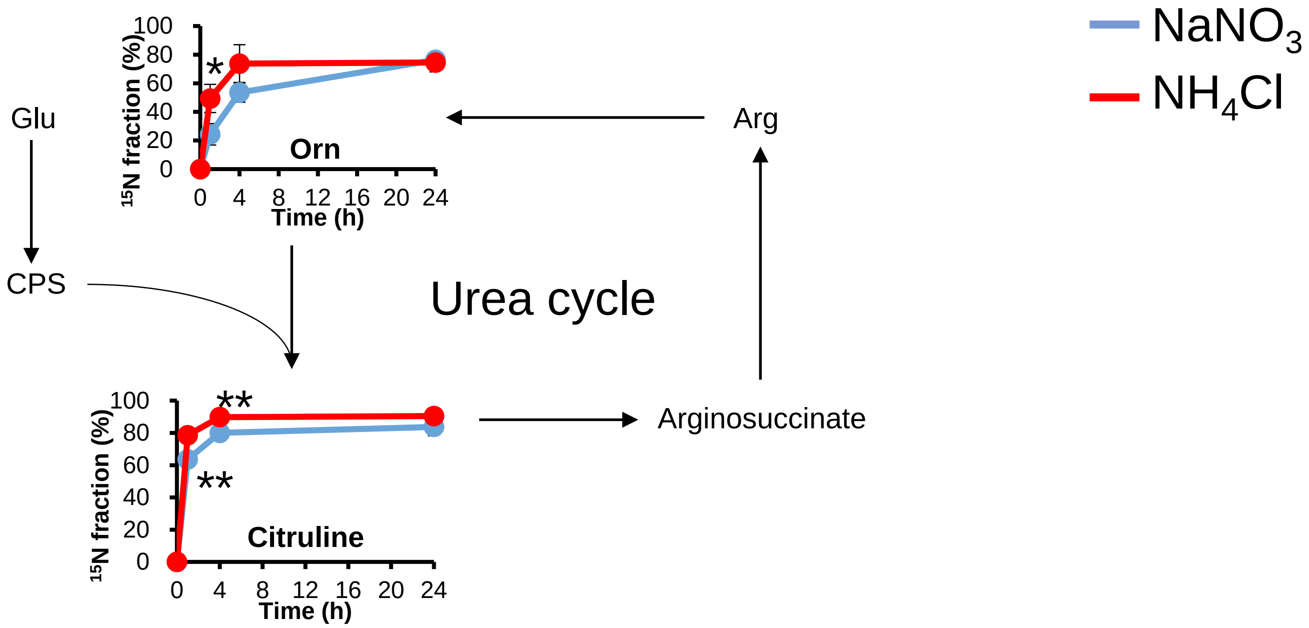

Figure S3 ^{15}N labelling rates of metabolites of the Urea cycle, ^{15}N labelling rates of the Urea cycle was compared at each time point. Blue lines, cultivation with $NaNO_3$; Red lines, cultivation with NH_4Cl.; Arg, Arginine; CPS, Carbamoyl phosphate; Glu, Glutamate; Orn, Ornithine.
